# Understanding Public Views about Air Quality and Air Pollution Sources in the San Joaquin Valley, California

**DOI:** 10.1155/2017/4535142

**Published:** 2017-04-02

**Authors:** Ricardo Cisneros, Paul Brown, Linda Cameron, Erin Gaab, Mariaelena Gonzalez, Steven Ramondt, David Veloz, Anna Song, Don Schweizer

**Affiliations:** Health Sciences Research Institute, University of California, Merced, CA, USA

## Abstract

The San Joaquin Valley of California has poor air quality and high rates of asthma. Surveys were collected from 744 residents of the San Joaquin Valley from November 2014 to January 2015 to examine the public's views about air quality. The results of this study suggest that participants exposed to high PM_2.5_ (particulate matter less than 2.5 microns in size) concentrations perceived air pollution to be of the worst quality. Air quality in the San Joaquin Valley was primarily perceived as either moderate or unhealthy for sensitive groups. Females perceived air pollution to be of worse quality compared to males. Participants perceived unemployment, crime, and obesity to be the top three most serious community problems in the San Joaquin Valley. Participants viewed cars and trucks, windblown dust, and factories as the principle contributors to air pollution in the area. There is a need to continue studying public perceptions of air quality in the San Joaquin Valley with a more robust survey with more participants over several years and seasons.

## 1. Introduction

Air quality is an important component to everyday life. The San Joaquin Valley (SJV) has some of the most polluted air in the nation [1]. This economically disadvantaged and ethnically diverse region [2–4] currently fails to comply with the federal standard for particulate matter 2.5 microns and smaller (PM_2.5_). This air pollutant is regulated under the National Ambient Air Quality Standards (NAAQS) to protect public health.

Air quality in the SJV contributes to the high number of emergency room visits and hospitalizations for a variety of respiratory and cardiovascular diseases including asthma, myocardial infarction, acute bronchitis, and pneumonia [5, 6]. The adverse impacts of the poor air quality in the SJV are not distributed equally. Recent research has shown that vulnerable populations bear a disproportionately large part of the health burden [2, 7]. Many efforts have been conducted by local air pollution control districts to educate the public about ways to reduce pollution in this area, including the provision of real time access to PM_2.5_ data that residents can use to plan activities and avoid being outdoors during the worst air pollution times [8]. Other efforts include the air quality flag program that provides public organizations such as schools with flags that show the air quality of the day [9, 10]. Avoiding exposure to air pollutants is integral to health and requires that the individual be aware when air quality is unhealthy and manage their personal exposure [11, 12]. However, little is known about the effectiveness of current communication efforts. Understanding the public's perception of air quality is an important aspect to aid the effort of risk communication and to develop tools to assist the public in avoiding exposure to air pollutants [13–15]. Studies regarding the public's perceptions of air quality in the SJV are lacking.

A survey was conducted with SJV residents to understand their sources of air quality information, perceptions of air quality, and behaviors related to air quality. The survey was developed and information collected through a partnership between a community advisory group and the University of California Merced's Health Sciences Research Institute. Other studies have been conducted from this data that focus on different questions [16]. However, the aim of this study was to assess public views and understanding of air quality and air pollution sources in the San Joaquin Valley.

## 2. Methods

### 2.1. Sample

**Figure 1:**
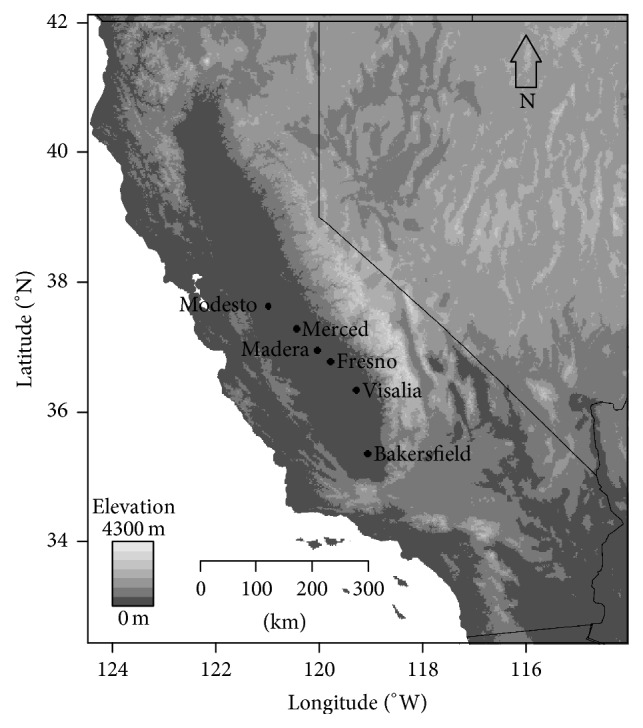
Air monitoring sites location map. The San Joaquin Valley is the area of the California Central Valley that lies south of the Sacramento River Delta (about 60 kilometers north of Modesto) and extends to Bakersfield.

The study area lies in the SJV of the California Central Valley and is shown in Figure 1. Residents of the SJV (*n* = 744) were surveyed in person (via community organizations and public locations) and from an online panel. The participants surveyed at community and public locations resided in Modesto and Merced, two cities in the north part of the SJV. An online survey (conducted through an online survey company) was available for participants from all locations in the SJV. The survey was conducted from November 2014 to January 2015. Further details of the survey methodology are available elsewhere [16]. Data were collected for 744 individuals. A total of 24 survey questions were asked, including demographic information: gender, age, education level, and zip code.

Ethical Institutional Review Board approval was obtained from the University of California, Merced.

### 2.2. Survey Method

Out of the 24 questions, three questions of the survey were used in this study (the other items are analyzed elsewhere [16]):

- “In the past month, what was the air quality like in other areas of the San Joaquin Valley?” (1 =* Very unhealthy, *2 =* Unhealthy, *3 =* Unhealthy for sensitive groups*, 4 =* Moderately healthy*, and 5 =* Good air quality*).
- “How serious of a problem is each of the following (car accidents, unemployment, crime, air pollution, infectious diseases (e.g., flu), forest fires, and obesity) in Modesto?” (1 =* Not at all serious*, 2 =* A little serious*, 3 =* Somewhat serious*, 4 =* Serious*, 5 =* Very serious*).
- “How much do each of the following (cars and trucks in the SJV, pollution from the bay area, farms and agriculture, factories, forest fires, windblown dust, construction, blowers and lawn mowers) contribute to air pollution in the San Joaquin Valley?” (1 =* Not at all*, 2 =* A little bit*, 3 =* Somewhat*, 4 =* A lot*, 5 =* Don't know*).

### 2.3. Air Quality Data

The air quality data was downloaded from the California Air Resources Board website. PM_2.5_ air data was used to assess the participants' exposure. After comparing to the one-, two-, and three-month PM_2.5_ averages, the two-month PM_2.5_ mean was used as the air quality exposure metric. The air quality exposure data were calculated based on the county of residence of the participant. The timeframe is the months before the date for each participant survey. The two-month PM_2.5_ mean concentrations were further grouped into three categories based on the United States NAAQS and the European Air Quality Standards: low, medium, and high. Low or good PM_2.5_ concentrations ranged from 0 to 12 *μ*g/m^3^ [17], medium or moderate ranged between 12 and 25 *μ*g/m^3^ [17, 18], and high or unhealthy concentrations were greater than 25 *μ*g/m^3^ [18].

### 2.4. Statistical Analysis

Descriptive statistics were used to describe the participants' demographics and responses to the questionnaire. Multivariable linear regression was used to determine the factors associated with participants' awareness of air quality in the SJV. Also, linear regression was used to examine if participant demographics could account for air pollution exposure level. Statistical significance was considered at the *p* < .05 level. Statistical analyses were performed using SPSS 20.

## 3. Results

### 3.1. Participant Characteristics and Air Pollution Exposure Levels

**Figure 2:**
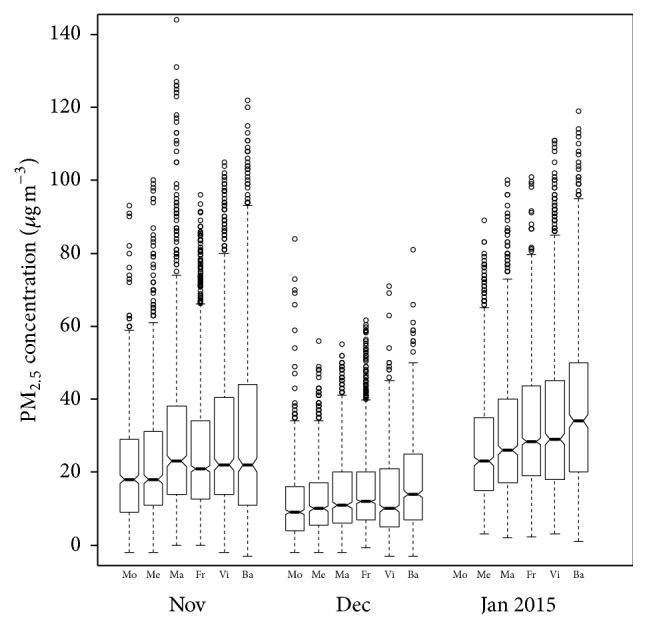
Air quality (PM_2.5_) in 6 locations (Modesto: Mo, Merced: Me, Madera: Ma, Fresno: Fr, Visalia: Vi, and Bakersfield: Ba) in the San Joaquin Valley from September 2014 to January 2015 ordered north to south. The survey was conducted November 2014–January 2015. Not all locations where the subjects resided are included in this figure. This is included to give the reader a view of air quality experienced by residents. The air in the SJV decreases in quality as you move south.

The participant demographics as well as outdoor air pollution exposure levels (PM_2.5_ level: high, medium, or low) are presented in Table 1. The majority of the participants (63%) were female. Close to half of the participants (51%) were over 40 years old. The general overall pattern is that PM_2.5_ concentrations, during the implementation of the survey, decrease as one moves south in the San Joaquin Valley (Figure 2). Only small percentage (7%) of our sample experienced low PM_2.5_ concentrations. The majority of our participants were exposed to medium levels (75%) and high levels (19%). The participants surveyed in Merced and Modesto experienced medium and low air pollution levels only. Very few (2%) of participants who responded via Internet experienced low air pollution levels and the majority (59%) experienced high air pollution exposure.

Results from a multivariate linear regression analysis of factors related to air quality exposure levels are presented in Table 2. Not surprisingly (see Table 1), location was found to be the only predictor (*β* = −.380, *p* < .001) of air quality exposure levels.

### 3.2. Perceptions of Air Quality

**Figure 3:**
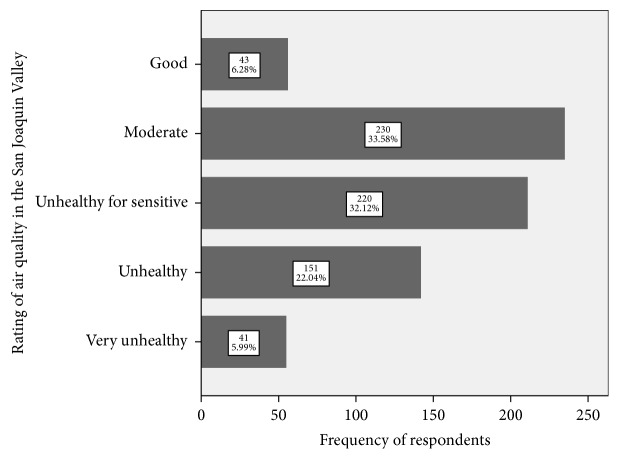
Participant rating of air quality in the San Joaquin Valley.

Figure 3 shows that 6% of the respondents perceived air quality in the SJV to be good; 34% responded that air quality was moderate; 32% answered that air quality was unhealthy for sensitive groups. Twenty-two percent of the respondents believed that the air quality was unhealthy. Only 6% responded that air quality was very unhealthy.

### 3.3. Factors Associated with Perceptions of Air Quality

Results from a multivariate linear regression analysis of factors associated with air quality perceptions are presented in Table 3. Air pollution exposure levels were found to be negatively associated (*β* = −.351, *p* = .001) with perception of air quality in the SJV. Participants who were exposed to higher PM_2.5_ levels ranked air quality worse when compared to participants who experienced lower PM_2.5_ levels. Females perceived the air quality to be worse compared to males (*β* = −.176, *p* = .030). Modality (web versus in person) and the location of the survey were associated (*β* = −.130, *p* = .038) with perception of air quality.

### 3.4. Relative Seriousness of Air Pollution

Table 4 presents information about the degree of concern about air quality framed in relation to other community problems (unemployment, crime, obesity, car accidents, infectious diseases, and forest fires). Table 4 shows that, for participants overall, the top three-ranking problems rated by the participants were unemployment, crime, and obesity with air pollution rated as the fourth most serious problem. The table also presents information about the perceived seriousness of air pollution when compared with other community problems categorized by air quality exposure level. For participants who were exposed to low and medium PM_2.5_ levels, air pollution ranked as the 4th most serious problem when compared to the other community problems. However, for participants who experienced high air pollution exposure levels, air pollution is ranked as the most serious problem, followed by unemployment, crime, and obesity.

### 3.5. Participants' Beliefs about Sources of Air Pollution

Table 5 presents the mean ratings of perceived contributions of sources to air pollution, along with the rankings of these means, and data on the actual air pollution sources (accounting for all reportable sources) gathered from the California Air Resources Board emissions data. Participants ranked farming and agriculture as the 5th most significant source of air pollution, which is discrepant with emissions data showing that farming and agriculture ranked 1st in actual emissions (Table 5). Most of the participants responded that the main contributor to air pollution in the SJV was cars and trucks which are the number 2 source of emissions (Table 5). Participants ranked windblown dust as the number two source (5th ranked actual emissions) and also ranked factories (6th ranked actual emissions) as number three.

## 4. Discussion

### 4.1. Air Pollution Exposure Levels and Perception and Comparison to Other Studies

This manuscript investigated factors that were associated with air pollution perception. Air pollution exposure level was found to be the most important factor. The higher the pollution exposure level, the worse the respondents' ranking of air pollution. This is surprising as many studies have found perceptions about air quality to be inaccurate [13, 19, 20]. However, our findings are similar to Claeson et al. [21] who found that air pollution exposure did not directly influence symptoms, but it did influence perceived pollution. The study by Claeson et al. [21] also suggests that perceived air pollution influences health risk perception which influences symptoms. It has also been suggested by Forsberg et al. [22] that people can detect air quality conditions below air quality standards. In that study, only nitrogen dioxide was correlated with annoyance related to air pollution and traffic exhaust fumes. It is worth noting that the participants in the Forsberg et al. [22] study have different sources and types of air pollutant exposures than people in the SJV. It is difficult to say that people can detect all types of air pollution, as they all cause different symptoms.

It is unclear how air pollution exposure levels influence the public's response. It is beyond the scope of this study to determine if direct experience with sensory cues (e.g., seeing or smelling dirty air) and impact (symptoms) of air quality influenced their response, or if it was due to outreach and continuing communication by the local air district that led to the participants' perceptions. A potential explanation for these results might come from the mass media. People rely heavily on information from mass media when it comes to environmental risks [23]. The high air pollution levels in the southern (Fresno and South to Bakersfield) part of the SJV receive a lot of media attention due to yearly reports [1].

In this study, females viewed air quality conditions to be worse compared to males. This result is similar to that of Lai and Tao [24], who found that women were more concerned about environmental hazards than men. This is different compared to the study by Howel et al. [25] who found no association with gender regarding perception of air pollution and health.

When investigating factors that were associated with air pollution exposure level, location was found to be the only predictor. These results are not surprising since PM_2.5_ exposure levels decrease north to south in the SJV.

### 4.2. Air Pollution versus Other Problems

When the participants were asked to rate air pollution in comparison to other problems (unemployment, crime, obesity, car accidents, infectious diseases, and forest fires), they rated air pollution as the number four problem out of the seven listed. The participants responded that unemployment, crime, and obesity are the top three problems. This creates a challenge for health education and communication. When the responses were categorized by air quality exposure level, those who experienced high exposure levels rated air pollution as the number one problem. This implies that perception of the intensity of poor air quality may relate to its prioritization.

### 4.3. Participant Knowledge of Air Pollution Sources

The participants were asked about their knowledge of the sources of air pollution in the SJV. The responses confirm that the participants' perceptions did not match the actual contribution source. It is apparent that participants underestimated farm and agriculture emissions which are the number one source contributor to air pollution in this area. Participants rank farms and agriculture as the number five contributor out of seven options. The participants' perceptions were closer in their ranking of cars' and trucks' contribution to air quality (which they ranked as number one), since the actual contribution of cars and trucks is ranked at number two.

### 4.4. Forest Fires Importance as Source of Air Pollution

Proximity to the Sierra Nevada forest ecosystem is another source of pollution that can impact people with asthma in this area at unpredictable times [26]. Increased drought and past land management fire suppression practices have removed fire from this fire-prone ecosystem. These practices have created conditions that make this area a current and future source of wildland fire smoke emissions that might further impact the SJV particularly via large high intensity wildfires not typical of Sierra Nevada forests [27, 28].

There has been much interest among policy makers, managers, and regulators from state and government agencies dealing with forest fire smoke impacts to communities of the SJV [27, 28]. Based on the participants' responses, it is obvious that forest fires are not a serious participant concern in the SJV. They rank forest fires as the least of the problems faced by SJV residents. When asked about their knowledge of contribution, they responded that forest fires were the number six contributor out of seven while they ranked 4th in actual emissions.

## 5. Conclusion

Air quality is of concern to residents of the San Joaquin Valley. There is a need to continue to monitor and study public perceptions of air quality in the SJV with a more robust survey that will have more participants and encompass several years. This is important if objectives of air quality and environmental management are to be achieved.

The following are our main conclusions:

1. Sixty-four percent of the participants responded that air quality was moderate and unhealthy for sensitive people.
2. Air quality exposure level was found to be the most important factor associated with perception of air pollution. Participants who were exposed to high PM_2.5_ levels perceived air pollution to be of worst quality.
3. When asked about the most serious problems in the SJV, the top three problems were unemployment, crime, and obesity. However, when categorized by air quality exposure level, those with high PM_2.5_ exposure responded that air pollution was the top problem in the SJV.
4. The top three air pollution contributors viewed by the participants were cars and trucks, windblown dust, and factories. The actual rank contribution is different compared to the participants' view, with farms and agriculture, cars and trucks, and pollution from the bay area being the actual top three contributors to air pollution.
5. Forest fires are the least of the participants' concern in the SJV.

## Figures and Tables

**Table 1:** Sample demographics including frequency (%).

	Location^1^			Total
	Modesto	Merced	Web^2^	
Gender				
Male	58 (22%)	106 (39%)	105 (39%)	269 (100%)
Female	186 (41.3%)	138 (30.7%)	126 (28%)	450 (100%)
Age				
≤40	92 (26%)	154 (44%)	105 (30%)	351 (100%)
>40	152 (41%)	90 (25%)	126 (34%)	368 (100%)
Education				
≤High school	127 (35%)	134 (37%)	104 (28%)	365 (100%)
≥College	117 (33.1%)	110 (31.1%)	127 (35.9%)	354 (100%)
Exposure level				
High	0 (0%)	0 (0%)	130 (100%)	130 (100%)
Medium	201 (38%)	235 (45%)	89 (17%)	525 (100%)
Low	41 (87%)	5 (11%)	1 (2%)	47 (100%)

^1^This also refers to the modality (in person versus Internet) of the survey.

^2^Participants who responded via the web reside in many locations in the San Joaquin Valley, including and not limited to the cities of Modesto, Merced, Madera, Fresno, Visalia, and Bakersfield.

**Table 2:** Factors related to air pollution exposure levels in the San Joaquin Valley.

	*β*	SE	*p* value
Intercept	2.845	.045	.000
Modality of survey/location	−.380	.018	.000
Age	.046	.029	.112
Female	.031	.030	.304
Education	.002	.029	.947

**Table 3:** Factors associated with perception of air pollution in the San Joaquin Valley.

	*β*	SE	*p* value
Intercept	4.363	.312	.000
Air pollution exposure levels	−.351	.101	.001
Female	−.176	.081	.030
Modality of survey/location	−.130	.063	.038
Education level	−.083	.079	.292
Age	−.002	.002	.358

**Table 4:** Participants' beliefs about the most serious community problems.

Problem	All data mean, SD, rank	Air pollution exposure level		
		High mean, SD, rank	Medium mean, SD, rank	Low mean, SD, rank
Unemployment	3.89, 1.113, 1	3.57, 1.131, 2	4.08, 1.056, 1	4.13, .924, 1
Crime	3.84, 1.084, 2	3.44, 1.172, 3	3.95, 1.059, 2	4.07, .831, 2
Obesity	3.73, 1.132, 3	3.36, 1.137, 4	3.78, 1.136, 3	3.98, 1.0, 3
Air pollution	3.55, 1.089, 4	3.63, 1.173, 1	3.56, 1.082, 4	3.71, .875, 4
Infectious diseases	3.01, 1.145, 5	2.88, 1.061, 6	3.03, 1.174, 5	3.05, 1.094, 6
Car accidents	2.98, 1.113, 6	2.91, 1.057, 5	2.93, 1.117, 6	3.23, 1.111, 5
Forest fires	2.55, 1.358, 7	2.70, 1.266, 7	2.43, 1.338, 7	2.53, 1.429, 7

**Table 5:** Participants' perception of contributors to air pollution in the San Joaquin Valley versus actual sources.

Problem	All data mean, SD, rank	Actual contribution^*∗*^ rank (%)
Cars & trucks	3.35, .808, 1	2 (17%)
Windblown dust	3.20, .872, 2	5 (8%)
Factories	3.19, .850, 3	6 (8%)
Pollution from bay area	2.99, .914, 4	3 (15%)
Farms and agriculture	2.98, .954, 5	1 (35%)
Forest fires	2.91, 1.014, 6	4 (9%)
Construction	2.64, .918, 7	8 (.82%)
Blowers and lawn mowers	2.61, .955, 8	7 (.98%)

^*∗*^Actual air pollution contribution calculated based on the 2012 estimated annual average emissions for the San Joaquin Valley Air Basin (Almanac Emission Projection Data, Published in 2013, downloaded from the Air Resources Board website).
